# Dissolved organic matter-mediated photodegradation of anthracene and pyrene in water

**DOI:** 10.1038/s41598-020-60326-6

**Published:** 2020-02-25

**Authors:** Siyu Zhao, Shuang Xue, Jinming Zhang, Zhaohong Zhang, Jijun Sun

**Affiliations:** School of Environmental Science, Liaoning University, Shenyang, 110036 China

**Keywords:** Environmental monitoring, Environmental sciences, Environmental chemistry

## Abstract

Toxicity and transformation process of polycyclic aromatic hydrocarbons (PAHs) is strongly depended on the interaction between PAHs and dissolved organic matters (DOM). In this study, a 125W high-pressure mercury lamp was used to simulate the sunlight experiment to explore the inhibition mechanism of four dissolved organic matters (SRFA, LHA, ESHA, UMRN) on the degradation of anthracene and pyrene in water environment. Results indicated that the photodegradation was the main degradation approach of PAHs, which accorded with the first-order reaction kinetics equation. The extent of degradation of anthracene and pyrene was 36% and 24%, respectively. DOM influence mechanism on PAHs varies depending upon its source. SRFA, LHA and ESHA inhibit the photolysis of anthracene, however, except for SRFA, the other three DOM inhibit the photolysis of pyrene. Fluorescence quenching mechanism is the main inhibiting mechanism, and the binding ability of DOM and PAHs is dominantly correlated with its inhibiting effect. FTIR spectroscopies and UV–Visible were used to analyze the main structural changes of DOM binding PAHs. Generally, the stretching vibration of N–H and C–O of polysaccharide carboxylic acid was the key to affect its binding with anthracene and C–O–C in aliphatic ring participated in the complexation of DOM and pyrene.

## Introduction

Polycyclic aromatic hydrocarbons (PAHs) are typical persistent organic pollutants with two or more fused benzene rings that are widely distributed in multi-media, such as atmosphere, water, sediment, snow, and biota^1–4^. PAHs undergo various natural reactions in environmental media, such as biodegradation, chemical transformation and photolysis^5,6^. Non-biological photodegradation related to solar irradiation may be an important process of degradation^7^. Related articles have reported that PAHs can be directly or indirectly photosensitized in aquatic environment^8^.

Dissolved Organic Matter (DOM) is a heterogeneous mixture of soluble substances commonly found in freshwater and marine environments. It consists of proteins, carbohydrates, hydrocarbons, humus and other components, accompanied by different functional groups^9^. DOM has a great influence on biodegradability, biological toxicity, migration and transformation properties of hydrophobic organic pollutants (HOCs) and metal elements in the environment, and its binding capacity depends on its origin and structural characteristics^10^. Studies have shown that DOM can promote and inhibit the photolysis of organic pollutants^11,12^. The promotion mechanism is mainly through indirect photolysis processes such as photosensitized oxidation reaction^13^. DOM has abundant aromatic rings, phenolic groups, hydroxyl groups, carboxyl groups and other chromophores, which are easily stimulated under light to produce simultaneously reactive oxygen species (ROS), such as hydroxyl radicals (OH) and singlet oxygen species (^1^O_2_), which then interact with organic pollutants to achieve their degradation effect^14^. The inhibition mechanism is mainly embodied in the abundant conjugated chromophore structure in DOM, which may be related to the photoshielding effect of organic pollutants competing to absorb light and the quenching effect of DOM on the excited state of pollutants^15^. This process is related to the bonding action between pollutant molecules^16^.

Polycyclic aromatic hydrocarbons (PAHs) can bind to DOM in aquatic environment. The binding mechanism has an effect on the ecological distribution of PAHs to a great extent^17^. There is no detailed report on its intrinsic influencing factors. Based on the complex mechanism of the effect of DOM on the photodegradation of PAHs, the inhibition mechanism of DOM on the photodegradation of PAHs is discussed from the perspective of binding capacity, which provides theoretical support for the development of PAHs degradation technology in water environment. Anthracene and pyrene are abundant in environmental media^18^. In this paper, anthracene and pyrene are selected as model materials for PAHs study to explore the mechanism of photodegradation of polycyclic aromatic hydrocarbons (PAHs) caused by four DOMs from land sources (LHA and ESHA) and water sources (SRFA and UMRN).

## Experimental Methods

### Reagents and chemicals

Anthracene (99.0%) and pyrene (97.0%) were from Shanghai McLean. Sodium hydroxide (NaOH) and acetonitrile were from Tianjin Damao Chemical Reagent Factory for analytical purity. Deionized water was prepared with Millipore Milli-Q system. Suwannee River fulvic acid Standard II (SRFA), Upper Mississippi River NOM (UMRN) are derived from water source DOM, lliott Soil Humic Acid Standard IV (ESHA), Leonardite Humic Acid Standard (LHA) are from land source DOM which were purchased from the International Humic Acid Association.

### Dissolved organic carbon and kinetic measurement

The content of DOC was determined by a Shimadou-5000 Total Organic Carbon Analyzers (Shimadou, Japan) after adjusting pH to neutrality. Anthracene and pyrene were quantitatively analyzed by high performance liquid chromatography (HPLC) equipped with an Agilent ZORBAX SB-C18 column as well as using a UV detector at 251 nm and 240 nm. The column temperature was controlled at 30 °C. The flow rate of a 90: 10 (V/V) mixture of acetonitrile: water as the mobile effluent was set at 1.4 mL/min.

### Spectrum measurements

UV–Visible spectra were recorded in the range of 190–600 nm on a Cary-50 ultraviolet-visible spectrophotometer (Varian Company) in a quartz colorimetric dish of 1 cm. The photolysis spectra of anthracene and pyrene in pure water and the mixed photolysis spectra of DOM and PAHs were obtained for qualitative analysis.

FTIR spectra were obtained on an IR Prestige-21 Fourier Transform Infrared spectrophotometer. DOM solid powder was obtained by drying a certain amount of DOM solution in oven at 30 °C. A mixture of 1 mg DOM and 300 mg KBr (spectral purity) was grinded and determined by infrared spectroscopy after 1:300 volume pressing. The result of determination has deducted the blank of infrared spectroscopy of potassium bromide tablets, and the data were performed using Origin 8.5.

The fluorescence intensity of anthracene and pyrene was collected by a Cary Eclipse EL0507-3920 (Varin Company, America) spectrophotometer. When DOM and PAHs coexist, they will interact and form non fluorescence PAHs-DOM complex. Chen *et al*.^19^ showed that this quenching effect is a static quenching mechanism. Based on stern Volmer equation, the combination degree of them is expressed. Determination of fluorescence quenching by constant wavelength synchronous fluorescence method. The excitation and emission slit width was 5 nm as well as the scanning speed is 1200.00 nm/min. The fluorescence spectrum of anthracene was set at excitation/emission wavelengths of 190 nm/350 nm and 250 nm/400 nm for pyrene. Delta interval is 1.0 and the factor was set at 30 for anthracene, 20 for pyrene. The stability of the instrument is tested by taking a baseline with a sample of distilled water. The DOM samples with DOC concentration of 0.66 mg/L, 1.32 mg/L, 1.98 mg/L, 2.64 mg/L and 3.3 mg/L were prepared. The 3.00 ml pipette of each sample concentration was transferred to the cuvette. The small amount of PAHs stock solution prepared by acetonitrile was added to the cuvette according to the solubility and fluorescence intensity, so that the final concentration of PAHs was 50 μg/L. The volume content of acetonitrile was less than 0.2% (V/V), which had no significant effect on the adsorption of PAHs.

### Photodegradation experiments

The illumination experiment was carried out in LY-GHX-V photochemistry reactor (Shanghai Lanyi Industrial Ltd.) and the experimental temperature was kept constant at 27 °C by circulating condensation tube. A 125 W high-pressure mercury lamp was used as the light source, and the UV radiation wavelength was more than 290 nm through a 290-nm cut-off wavelength filter to simulate sunlight. Three parallel samples were set in each group, and the control experiment was set in darkness condition at the same time. The illumination time was 3 hours, and a group of samples were taken every 0.5 hours. Anthracene and pyrene are in a 20 ml quartz tube. Quartz is the best material for illumination, it is transparent to the radiation light (UV-A) reaching the surface of the solution. The temperature effect of the quartz tube during illumination can be neglected.

PAHs are hydrophobic. PAHs stock solution (200 mg/L) was prepared in acetonitrile and stored in amber borosilicate vials at 4 °C in refrigerator. DOM was prepared with deionized water and TOC concentration was 3.3 mg/L. The pH of sample solution was adjusted to neutral (pH = 7). DOM solution (PAHs = 50 ug/L, DOM = 3.3 mg/L) was added to PAHs for photolysis of DOM and PAHs. The sample was dissolved by ultrasound for 10 minutes and stored in 4 °C condition.

## Results and Discussion

### Photolysis kinetics of Anthracene and Pyrene

**Figure 1:**
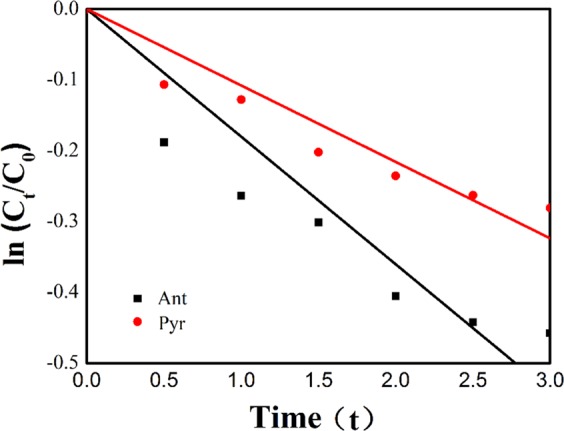
Photolysis Kinetic Fitting Curves of Anthracene and Pyrene.

Under illumination conditions, anthracene and pyrene both undergo direct photodegradation and obeys the first-order kinetics model^20^. The first-order kinetic equation was tried to have model fitting to the plot which used ln (C_t_/C_0_) as x axis and t (min) as y axis. The rate constant (k) of the apparent reaction was obtained from the plot (Eq. (1)) and the half-life (t_1/2_) was calculated from the Eq. (2):

1 $$-\,\mathrm{ln}(\frac{C{\rm{t}}}{Co})=kt$$

2 $${t}_{\frac{1}{2}}=\frac{0.693}{k}$$

where t is the photolysis time, C_t_ is the concentration of PAHs in the photolysis solution at time t, C_0_ is the concentration of PAHs in the initial photolysis solution, K is the apparent photolysis rate constant of PAHs. As shown in Fig. 1, the photolysis rate constants of anthracene and pyrene are 0.1801 (r = 0.9596) and 0.1079 (r = 0.9696), respectively. The extent of degradation of anthracene and pyrene can reach 36% and 24%, which shows that anthracene is easier to photolysis in water environment. Compared with the previous ice phase data, the extent of degradation of PAHs is three times higher than that of ice phase condition, which indicates that water phase condition is more conducive to the photodegradation of anthracene and pyrene. This may be due to the indirect photolysis of PAHs and hydroxyl radicals in water, which accelerates the transformation of PAHs. Neff and Fasnacht *et al*.^21,22^ also showed that PAHs and hydroxyl radicals (·OH) undergo both direct photodegradation and indirect photooxidation in water. Other studies have shown that hydroxyl radicals in ice and snow environments have little effect on the photodegradation of phenanthrene, pyrene and fluoranthene, but not the main mechanism. The concentration of anthracene and pyrene did not change significantly under dark conditions, which indicated that anthracene and pyrene were not easily hydrolyzed in water environment, and photodegradation was an important degradation mode.

## Effect of DOM on the photolysis of Anthracene and Pyrene

**Figure 2:**
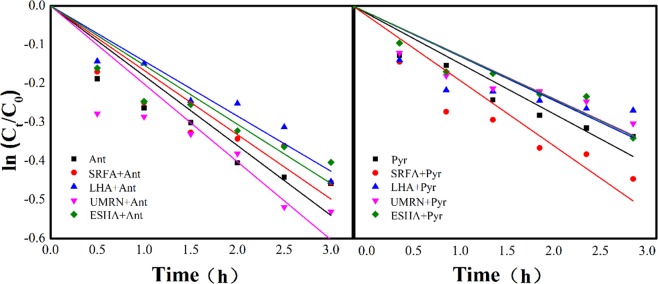
Fitting curve of PHAs photolysis kinetics before and after DOM addition.

Figure 2 showed the photolysis kinetics of anthracene and pyrene in pure water and four DOM solutions. It was found that the effects of four DOMs on the photolysis of anthracene and pyrene showed two mechanisms. ESHA and LHA inhibited the photolysis of anthracene and pyrene, and their effects were similar. UMRN and SRFA had different effects on the photolysis of anthracene and pyrene. UMRN promoted the photolysis of anthracene and pyrene, and inhibited the photolysis of pyrene. SRFA, contrary to UMRN, promoted the photolysis of pyrene. The apparent photolysis rate constants of anthracene in SRFA, LHA, UMRN and ESHA were 0.15, 0.14, 0.20, 0.17, pyrene 0.09, 0.09, 0.09 and 0.14, respectively.

It is generally believed that the inhibition of photolysis of PAHs by DOM is mainly due to its photoshielding effect. The ratio of the experimental value to the theoretical value can be used to estimate the degree of the photoshielding effect. If the ratio is less than 1, it indicates that there are other mechanisms besides the photoshielding effect, such as the quenching effect of DOM on the excited state of pollutants; if the ratio is equal to 1, it indicates that the photoshielding effect is suppressed. The main mechanism of photolysis of polycyclic aromatic hydrocarbons. Some formulas for the quenching effect of the actual value (*S*_λ_′) and the theoretical value (*S*_λ_) of the optical shielding are expressed by (3) (4) (5)^8^.

3 $$S\lambda =\frac{1-{10}^{-(\alpha \lambda +\varepsilon \lambda {{C}}_{{PAHs}}){l}}}{2.303(\alpha \lambda +\varepsilon \lambda {{C}}_{{PAHs}}){l}}$$

4 $${S}_{\lambda }{\prime} =\frac{K{\rm{obs}}}{Kd}$$

5 $$Q\lambda =1-\frac{{S}_{\lambda }{\prime} }{S\lambda }$$

As show in Table 1, it can be seen that the ratio of actual value to theoretical value of four kinds of DOM is less than 1, which indicates that besides the effect of optical shielding, the quenching effect of DOM inhibits the photodegradation of PAHs. The quenching effect of DOM from two sources on pyrene is different, and the fluorescence quenching effect of DOM from water source on anthracene and pyrene is stronger. John *et al*.^23^. showed that the quenching effect was mainly related to the binding ability of DOM and pollutant molecules. The effect of binding capacity on the photolysis of PAHs will be analyzed in detail in the next section.

**Table 1:** Actual and theoretical values of four kinds of DOM light shielding and quenching effect.

Solution Category	K_DOM_(h^−1^)^a^	S_λ_	*S*_λ_′/S_λ_	*Q*_λ_
**Anthracene**				
UMRN	0.007	0.9925	0.9637	0.0363
SRFA	0.013	0.9951	0.9268	0.0732
ESHA	0.022	0.9695	0.8745	0.1255
LHA	0.034	0.9766	0.8079	0.1921
**Pyrene**				
UMRN	0.014	0.9890	0.8687	0.1313
SRFA	0.007	0.9917	0.9317	0.0683
ESHA	0.011	0.9662	0.8940	0.1060
LHA	0.011	0.9733	0.8989	0.1011

^a^Represents the observed rate constant of anthracene and pyrene photodegradation in the presence of DOM.

## Analysis of Binding Ability of DOM with Anthracene and Pyrene

K_DOM_ was determined by fluorescence quenching method. Polycyclic aromatic hydrocarbons (PAHs) are fluorescent substances, which emit fluorescence at a specific wavelength. When DOM binds to PAHs, some of the fluorescence properties will be inhibited. Therefore, the apparent binding constants of PAHs to DOM can be determined indirectly by the degree of fluorescence quenching. The binding coefficient K_DOM_ between PAHs and DOM is based on the following reaction equation^24^:

6 $$PAHs(aq)+DOM(aq)\to PAHs-DOM(aq)$$

7 $$K=\frac{[PAHs-DOM]}{[PAHs]\times [DOM]}$$

8 $$\frac{{[PAHs]}_{t}-[PAHs]}{[PAHs]}=K\times [DOM]$$

[PAHs]_t_ is the initial concentration of PAHs, mg/L. In theory, the fluorescence intensity is proportional to the concentration of fluorescent substances, so formula (8) can be written as Stem-Volmer equation.

9 $$\frac{{F}_{0}}{f}=1+K[DOM]$$

F_0_—— Initial Fluorescence Intensity of Fluorescent Substances.

$f$——Fluorescence intensity of fluorescent substances adsorbed by DOM.

[DOM] —— Concentration of DOM, mg · C/L.

K_DOM_—— DOM Combined with PAHs Constant, L/kg.

**Figure 3:**
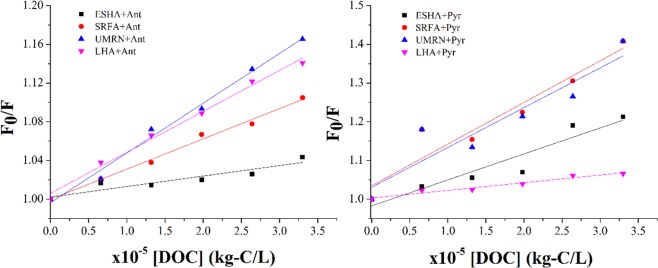
Stem-Volmer plots of ESHA, SRFA, UMRN and LHA.

According to Stem-Volmer, K_DOM_ is the slope of the equation. It can reflect the balance of pollutants between DOM and water solution and the law of migration and transformation. It is an important index to characterize the behavior of PAHs in water environment^25^. Relevant articles show that^26^, K_DOM_ is positively linearly correlated with octanol/water partition coefficient and hydrophobicity. As shown in Fig. 3, the Stem-Volmer diagrams of four DOMs are linearly correlated. The binding of DOM with anthracene and pyrene to form complexes is mainly due to hydrophobic interaction. The binding ability of four DOMs to anthracene was in turn: UMRN > LHA > SRFA > ESHA, and the binding constants were 0.0471 (r = 0.97), 0.042 (r = 0.98), 0.029(r = 0.98) and 0.010 (r = 0.84), respectively. The binding ability with pyrene is SRFA > UMRN > ESHA > LHA and the constant of them are 0.164(r = 0.89), 0.108(r = 0.84), 0.034 (r = 0.88) and 0.019 (r = 0.95). From the apparent photolysis rate constants of anthracene and pyrene, the binding constants of DOM and PAHs affect the photolysis rate of polycyclic aromatic hydrocarbons to a certain extent. The stronger the binding capacity was, the stronger the inhibition was. But there is no complete correlation. Pearson correlation analysis of K_DOC_, Q and photolysis rate constant K showed that Pearson correlation is negative as shown in Table 2, indicating a negative correlation between binding capacity and quenching effect. The weaker the binding capacity, the stronger the quenching effect. The stronger the binding ability was, the greater the influence on photodegradation was. It can be said that the stronger the binding ability between DOM and PAHs, the stronger the inhibition. In addition, it has been reported that there are a large number of methyl isoelectronic acceptors in the hydrophobic structure of DOM, accepting electrons from PAHs, which further enhance the binding of this part of the structure to PAHs^27^.

**Table 2:** Correlation analysis parameters between the binding capacity of DOM to anthracene and pyrene and quenching effect.

Analysis object	P	Pearson correlation
K_DOC_ and *Q*_λ_	0.0816	−0.099
*Q*_λ_ and k	0.0592	0.225
K_DOC_ and k	0.0165	−0.543

## Ultraviolet-Visible Spectrum Analysis

**Figure 4:**
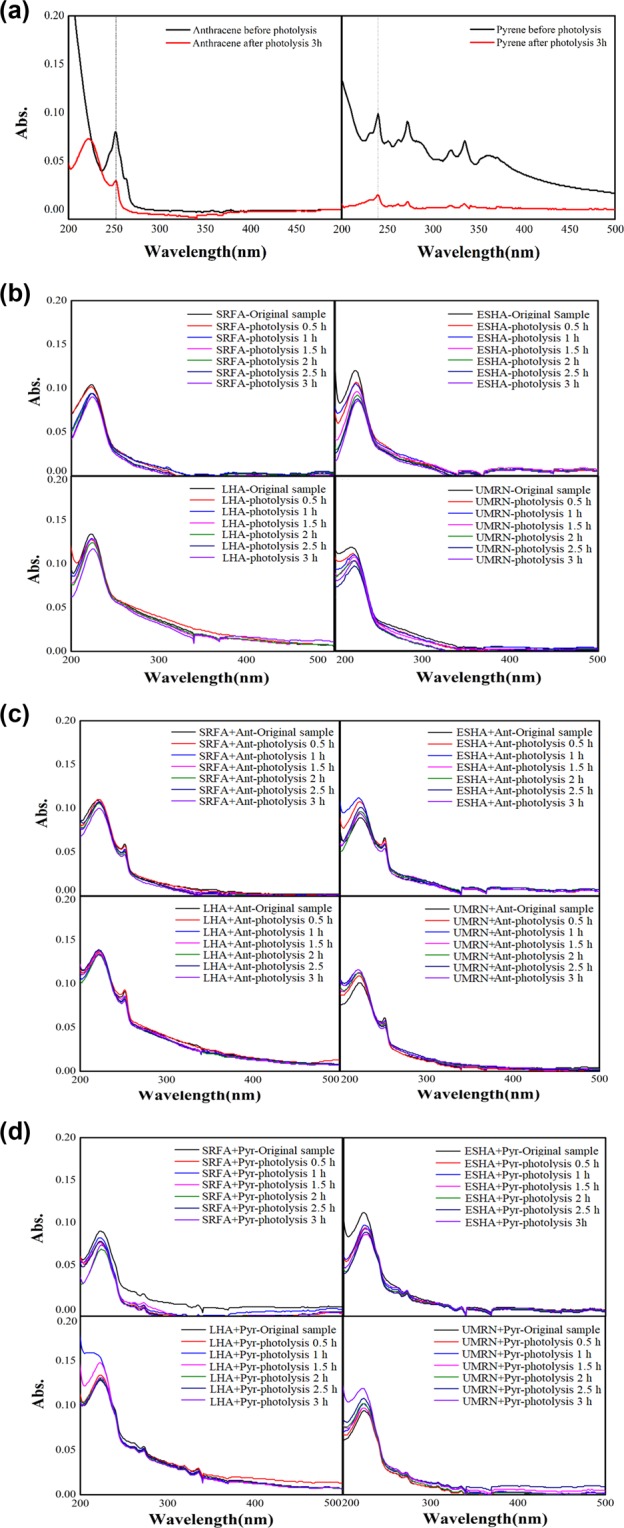
**(a)** Photolysis Ultraviolet Spectra of Anthracene and Pyrene. **(b)** Photolysis ultraviolet of DOM. **(c)** Photolysis ultraviolet spectra of mixtures of DOM and Anthracene. **(d)** Photolysis Ultraviolet Spectra of DOM and Pyrene.

UV absorption spectrum is usually used to study the structure of complex^28^. Figure 4 (a) is a full wavelength scanning of anthracene and pyrene at 200-600 nm. After 3 hours of illumination, the absorbance of anthracene and pyrene at the maximum ultraviolet absorption peak decreased significantly, the shoulder peak disappeared at 261 nm and 250 nm of pyrene, and other characteristic peaks weakened to varying degrees, which indicated that illumination had a great influence on anthracene and pyrene. Figure 4 (b) shows the UV photolysis of four DOM. The UV spectrum of DOM is usually a broad UV absorption peak with λ < 400 nm, which is produced by the internal vibration and rotation of many chromophores and molecules in humus and the interaction between molecules. From the electronic transition point of view, the UV absorption peak of DOM is λ > 240 nm, which is caused by the transition of aromatic matters with electron transfer. It has the characteristic electronic spectrum of aromatic matters and is the main source of DOM UV absorption^29–31^. As shown in Fig. 4 (a), four kinds of DOM have light absorption in the wavelength range of 230-400 nm. The absorbance of SRFA, ESHA, LHA and UNRN decreased in different degrees after 3 hours of illumination, and the position of UV absorption peak changed. E2/E3 is the ratio of DOM absorbance at 250 and 365 nm, can be used as an indicator of the structural difference of humic substances^32,33^. The E2/E3 values of SRFA, ESHA, LHA and UMRN before and after photolysis were 30 to 25, 15 to 31, 2.90 to 4.07 and 10.67 to 12, respectively. It showed that under the light condition, the macromolecular matter of DOM may be partially decomposed into micro molecule matters, and the structural change degree and position of DOM from different sources are different. With the increase of illumination time, the absorption platform of SRFA and UMRN between 300–400 nm gradually slowed down. Kieber *et al*.^34^ found in the photodegradation experiment of DOM that this may be related to the partial mineralization of organics or the formation of less absorbed organic compounds. When PAHs and DOM are mixed for photodegradation (Fig. 4(c,d)), the absorbance of anthracene and pyrene at the characteristic peak is significantly reduced. The absorbance at 270–280 nm is usually due to π - π* electronic transition of aromatic structure, such as phenolic compounds, benzoic acid and aromatic polycyclic hydrocarbons^35^, DOM may bond with PAHs to produce substances with weak UV absorption capacity, especially the UV absorption peak at 272 nm. The UV absorption peak of pyrene itself appears at 248 nm (Fig. 4(d)), which indicates that the combination of DOM and pyrene leads to the phenomenon of light masking. The functional group information involved in the combination of DOM and PAHs will be discussed in the infrared analysis section.

## Infrared Spectrum Analysis

**Figure 5:**
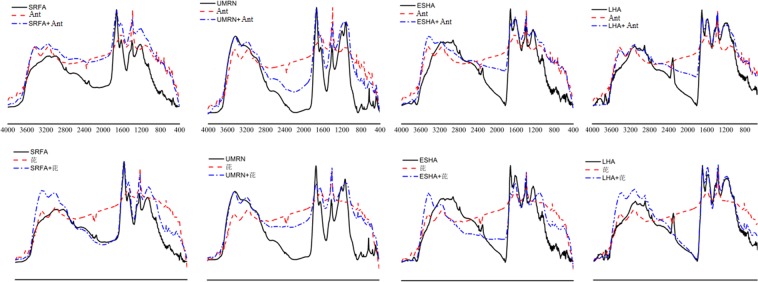
Infrared Spectra of DOM and Anthracene and Pyrene.

It can be seen from the Fig. 5 that anthracene has three main absorption peaks, which are located at 3500–3000 cm^−1^, 1639–1385 cm^−1^ and 668 cm^−1^, respectively. The strong absorption peaks at 3500–3000 cm^−1^ and 668 cm^−1^ are related to the stretching vibration of C–H on aromatic rings, 1639–1385 cm^−1^ is related to the stretching vibration of C=C on aromatic rings, and the absorption characteristic regions of aromatic hydrocarbons are mainly located at these threepositions^36^. The similar infrared spectra of ESHA and LHA indicate that their skeleton structures are basically close and their functional groups are different. According to Morrison *et al*.^37^ and Kaisertet *et al*.^38^, 3532 cm^−1^ and 3389 cm^−1^ in DOM molecule are two peaks of N–H stretching vibration, 2960 cm^−1^–2850 cm^−1^ are C-H stretching vibration of aliphatic methylene, 1740 cm^−1^–1706 cm^−1^ are C=O stretching vibration absorption peaks of aldehydes and ketones, and 1640 cm^−1^–1625 cm^−1^ are carboxylic acids with benzene ring, olefin C=C and intermolecular or intramolecular hydrogen bonds. The C=O stretching vibration peaks in benzene ring are C=O, 1384 cm^−1^ is N–O stretching vibration peak of nitrate, 1250 cm^−1^–900 cm^−1^ is C–O stretching vibration absorption peak of polysaccharide carboxylic acid and esters, and 870-640 cm^−1^ is characteristic peak of out-of-plane bending vibration absorption peak of benzene ring C–H. ESHA and LHA 3731 cm^−1^, 2920–2850 cm^−1^, 1640–1625 cm^−1^, 870–640 cm^−1^ have absorption peaks, indicating that DOM molecule contains aromatic rings, olefins and aliphatic methylene components, and 1250-900 cm^−1^ has moderate absorption peaks indicating polysaccharide carboxylic acid. 672–553 cm^−1^ is the stretching vibration of hydroxyl group.

After the combination of anthracene and ESHA, the absorption peak shifted to 3400 cm^−1^ at 3446 cm^−1^, and the intensity of absorption peak increased. At 3400 cm^−1^, the characteristic peak of DOM -NH stretching vibration increased, and the position intensity of absorption peak increased, and the C–H bond on anthracene aromatic ring changed. At the same time, new absorption peaks appeared at 1710 cm^−1^ and 1213 cm^−1^, and higher absorption peaks appeared at 1234 cm^−1^. After the combination of anthracene and LHA, 3435 cm^−1^ shifted to short wavelength direction, 1712 cm^−1^ strong absorption peaks appeared, 1385 cm^−1^ medium intensity absorption peaks appeared, 1172 cm^−1^ absorption peak intensity increased, indicating that the combination of terrestrial DOM and PAHs was mainly reflected in the stretching vibration of N–H and the stretching vibration of C–O of polysaccharide carboxylic acid in DOM. The binding of DOM and anthracene might have NH-π force. Smidt *et al*.^39^ reported that NH-π can be formed between indole and benzene ring compounds. The binding absorption peaks of SRFA and anthracene were strongly absorbed at 1640–1625 cm^−1^, and C–O vibration absorption peaks were observed at 1214 cm^−1^. A new absorption peak was observed at 1140 cm^−1^–1080 cm^−1^ for the binding of UMRN and anthracene, which was related to the S–O stretching vibration of inorganic sulfates. In contrast, the spectra of terrestrial DOM combined with anthracene changed greatly, and the effect on anthracene was stronger.

In addition, the infrared spectra of pyrene binding to DOM are similar to those of anthracene. There are strong absorption peaks at 1619 cm^−1^ and 1383 cm^−1^, weak absorption peaks at 3500 cm^−1^–3000 cm^−1^ and 615 cm^−1^, corresponding to the stretching and bending vibration of C-H on benzene ring and the stretching vibration of C=C on benzene ring. Four kinds of infrared spectra of DOM combined with pyrene showed a slight shift to short wavelength at 3500 cm^−1^–3000cm^−1^. ESHA, LHA, SRFA and UMRN showed new absorption characteristic peaks at 1075 cm^-1^, 1204 cm^−1^, 1176 cm^−1^ and 1068 cm^−1^, respectively, indicating that there might be C–O bond formation of polysaccharide carboxylic acid and C–O–C asymmetric stretching vibration of ether bond in aliphatic compounds aliphatic rings^40^. The absorption peaks of four kinds of DOM appeared at 1384 cm^−1^, and the N–O stretching vibration of nitrate existed, which indicated that the C–O functional groups of polysaccharide carboxylic acid were involved in the formation of complexes.

## Conclusions

Photodegradation is the main degradation mode of anthracene and pyrene in water environment. The effects of DOM from different sources on anthracene and pyrene can be divided into promotion mechanism and inhibition mechanism. The inhibition mechanism of DOM on PAHs is related to its light shielding effect and quenching effect. The stronger the binding ability of DOM to PAHs, the stronger the quenching effect and the stronger the inhibition effect on PAHs. The binding of terrestrial DOM with anthracene mainly involves the contractive vibration of N–H and the contractive vibration of C–O of polysaccharide carboxylic acid. The binding of water DOM with anthracene is related to the contractive vibration of N–H and the S–O contractive vibration of inorganic sulfate. The binding of four kinds of DOM with pyrene is mainly caused by the asymmetric stretching vibration of C–O–C bonds in aliphatic rings. At the same time, C–O functional groups are formed.

## Data availability

The datasets generated during and/or analyzed during the current study are available from the corresponding author on reasonable request.
